# Modulated evaluation metrics for drug-based ontologies

**DOI:** 10.1186/s13326-017-0124-2

**Published:** 2017-04-24

**Authors:** Muhammad Amith, Cui Tao

**Affiliations:** School of Biomedical Informatics, University of Texas Health Science Center, Fannin Street, Houston, Texas USA

**Keywords:** Ontology, Ontology evaluation, Quality assessment, Drug ontologies, Semiotics, Metrics, Knowledgebases

## Abstract

**Background:**

Research for ontology evaluation is scarce. If biomedical ontological datasets and knowledgebases are to be widely used, there needs to be quality control and evaluation for the content and structure of the ontology. This paper introduces how to effectively utilize a semiotic-inspired approach to ontology evaluation, specifically towards drug-related ontologies hosted on the National Center for Biomedical Ontology BioPortal.

**Results:**

Using the semiotic-based evaluation framework for drug-based ontologies, we adjusted the quality metrics based on the semiotic features of drug ontologies. Then, we compared the quality scores before and after tailoring. The scores revealed a more precise measurement and a closer distribution compared to the before-tailoring.

**Conclusion:**

The results of this study reveal that a tailored semiotic evaluation produced a more meaningful and accurate assessment of drug-based ontologies, lending to the possible usefulness of semiotics in ontology evaluation.

## Background

Given a scenario where a researcher is to choose two distinctly independent ontologies that cover a specific domain, how would the researcher know which is suitable between the two? Or given another scenario where a knowledge engineer is developing an ontological knowledgebase, how would she evaluate the quality of the ontology and know what to measure? This paper aims to provide a direction in the area of ontology evaluation using a system shaped by the theory of semiotics – the study of meaning for signs and symbols, specifically for biomedical ontologies.

Biomedical ontologies have influenced medical research with the impact and efforts of the Gene Ontology [1], UMLS [2], SNOMED [3], etc. It is assumed that ontological knowledgebases for biomedicine will grow to cover many other sub-domains. Already, an NIH-funded initiative, the National Center for Biomedical Ontologies (NCBO), exist to provide tools and hosting support for ontologies, and an active community of biomedical researchers formed the Open Biomedical Ontologies (OBO) Foundry [4] for rigorous standards for biomedical ontologies.

Semiotics is formally defined as the “the study of signs and symbols and how they are used” [5]. Abstractly, an ontology, with its terms and labels, can be a symbolic representation or signifier of a domain space that describe a physical manifestation of the real world. However, framing the ontology domain in semiotics is inherently common. While touching upon the three branches of semiotics, Sowa made a philosophical-oriented explanation of how the study of signs relate to 1) the syntax of an ontology (*syntactic*), 2) the meaning and logic derived from the syntax (*semantics*), and 3) the users or agents that interpret or utilize the signs (*pragmatics*) [6]. Approaching ontology evaluation from the semiotic frame is a natural choice to assess the overall craftsmanship of the ontology.

Our research questions in this study focus on 1) whether a semiotic-based approach for ontology evaluation can provide meaningful assessments for biomedical ontologies, and 2) whether this approach can be tailored for specific types of ontologies to provide more accurate quality assessments. The use-case focus will be drug-related ontologies hosted on the National Center for Biomedical Ontology BioPortal.

### National Center of Biomedical Ontologies

The National Center for Biomedical Ontologies (NCBO) is a NIH-funded program to provide support tools, and a repository to store a wide range of ontologies from the biomedical field. Based on a random survey sample of selected ontologies conducted from August 2015 (*n*=200), the authors of this paper searched for published studies that coincided with the development and the release of the ontology. The outcome of this brief survey revealed that most of the ontologies from this sample did not have any documented evidence of any evaluation (*n*_1_=183). A relatively small number had some evidence of any evaluation (*n*_2_=17). We can surmise that there is a need for evaluation, and that many biomedical ontologies lack any formal evaluation.

Also from our review, we noted that if there was any documented evidence of evaluations, the evaluation focused on a specific type of assessment. Some report statistical-related information denoting the number of ontological elements (classes, properties, etc.) or structural elements (depth, breadth, etc.). Others reported query-based or competency questions-driven approaches to evaluate the degree to which the ontology fulfills a use-case. A few utilized subject matter experts to review the general content, and a few measured some specific application tasks. Broadly, ontology evaluation appears to be diversified and focused.

### Semiotic Framework for Ontology Evaluation

While there are no agreed standard for ontology evaluation, researchers have proposed various evaluation approaches, such as, metric-based evaluation [7, 8], coverage of domain [9, 10], use-case and requirement assessment [11], and comparison with other ontologies sharing the same domain [7, 12]. In this study, we applied a metrics-based method that is rooted in semiotic theory, and also tailored this method to compare with ontologies in a similar domain.

A semiotic framework approach for ontology evaluation [13] was proposed by Burton-Jones, et al, nearly a decade ago when DAML-based ontologies were in existence. Reorganizing the intrinsic and extrinsic views of ontologies, it aims to be a holistic, domain-independent, and customizable approach to evaluate a wide range of ontologies by framing it in semiotic theory. Scores are denoted by the pillars of semiotics – *pragmatic*, *syntactic*, and *semantic*. An additional score, *social*, denotes an ontology’s ranking with other ontologies in a community. We intend to apply this metric suite for this study. To derive some of those scores, external software, like a triple store or WordNet-based APIs, are required. Detailed discussion of the scoring metric is provided here at [13], but we will summarize the aspects of the metric in the following sub-sections. The Eq. (1) below describe the overall quality evaluation score based on the four scores.

1 $$\begin{array}{@{}rcl@{}} Q=w_{q_{1}}*S + w_{q_{2}}*E + w_{q_{3}}*P + w_{q_{4}}*O \end{array} $$

The scores range from 0 to 1, where 1 is the highest and 0 is the lowest. Each of them weighted equally, yet there are mechanisms to tailor the weights to provide more influence of a certain aspect or diminish its influence. For example, if one were to measure the quality of an ontology that serves as a hierarchal terminology of terms, then it would make sense to decrease the weight of the syntactic score since it may under-utilize ontology features. (2-5) describe the underlying derivatives of the individual scores and their sub-scores.

#### Syntactic

Encoded ontologies enable machines to process and interpret the knowledge embedded in the knowledgebase. The *syntactic* score (2) describes the encoded readability of the ontology. *Lawfulness* (*S* *L*) and *richness* (*S* *R*), sub-scores of the *syntactic* score, represent conformity of the syntax, and the utilization of the ontology syntactic features. *S* *L* is calculated by the number of axiom-level violations based on the OWL 2 standards over the total number of axioms. The figures can be obtained using the OWL API. *S* *R* is based on the number of ontological features utilized over the total number of ontological features.

2 $$\begin{array}{@{}rcl@{}} S=w_{s_{1}}*SL + w_{s_{2}}*SR \end{array} $$

#### Semantic

Terms or labels are one of the fundamental building blocks of ontological knowledgebases. The *semantic* score (3) rates the terms’ understandability from 3 sub-scores. *Interpretability* (*E* *I*) rates the ontology’s terms from calculating the percentage of terms with at least one word sense. *Consistency* (*E* *C*) denotes the percentage of terms that are uniform among the ontology or lack of duplicate terms (number of duplicates over total number of terms), and *clarity* (*E* *A*) reveals how each term in the ontology are ambiguous based on the average number of word senses for each term (the average word sense per term over the number of terms).

3 $$\begin{array}{@{}rcl@{}} E=w_{e_{1}}*EI + w_{e_{2}}*EC + w_{e_{3}}*EA \end{array} $$

#### Pragmatic

*Pragmatic* score (4) is composed of three sub-scores, which includes *comprehensiveness* (*P* *O*), *accuracy* (*P* *U*), and *relevancy* (*P* *R*). *Comprehensiveness* scores an ontology’s domain coverage based on the percentage number of instances, classes, and properties of the ontology to a group of ontologies. *Accuracy* and *relevancy* are unique. The former requires domain experts to review and assess the veracity of facts evoked from the ontology – percentage of truthful statements. *Relevancy* varies and depends on possible use-case of the ontology. For example, if evaluators are concerned about the ontology’s ability to preform semantic-based searches, then a percentage of how successful queries is recorded as the *relevancy* score. (4) represents the composition of the *pragmatic* score.

4 $$\begin{array}{@{}rcl@{}} P=w_{p_{1}}*PO + w_{p_{2}}*PU + w_{p_{3}}*PR \end{array} $$

#### Social

While not particularly related to semiotics, the *social* score (5) is an assessment of the ontology’s “standing” in comparison with other ontologies. The *authority* (*O* *T*) sub-score is based on the percentage number of links that the ontology extends with other ontologies and the *history* (*O* *H*) sub-score is the percentage based on the number of times the ontology was accessed.

5 $$\begin{array}{@{}rcl@{}} O=w_{o_{1}} * OT + w_{o_{2}} * OH \end{array} $$

In the following sections, we will describe the methodology for utilization of the metric suite, and briefly discuss drug-based ontological datasets. Afterward, the paper will discuss the results and impact of our results for drug-based ontologies.

## Methods

We experimented with a set of biomedical ontologies from NCBO Bioportal that have the most visits (based on September 2015 data), according to the NCBO website. A total of 66 ontologies were sampled, but 2 were removed due to issues with the serialization of the files. With the 64 we calculated an aggregation of the scores and produced the basic statistics (mean, median, etc.) from them. Table 1 shows the results of this effort.

**Table 1:** NCBO sample aggregate scores

Quality	Mean	Std. Deviation	Min	Max
Syntactic	.64	.14	.18	.85
Lawfulness	.92	.16	.27	1
Richness	.36	.18	.07	.69
Semantic	.88	.15	.09	.99
Interpretability	.88	.14	.01	1
Consistency	.84	.40	-.17	1
Clarity	.96	.13	.14	1
Pragmatic	.02	.07	0	.52
Comprehensiveness	.02	.07	0	.52
Social	.02	.02	0	.13
History	.02	.02	0	.13
**Overall Score**	**.39**	**.05**	**.21**	**.48**

We also gathered a set of drug-related ontologies (See Drug Ontologies) and preformed the same aggregation scoring (Table 2). In addition, we also examined each of the scores to understand the quality of each drug ontology and the whole set in general. Finally, we tailored the metrics rooted on strengths and weakness of the drug ontologies, and compared the non-tailored and tailored aggregation.

**Table 2:** Drug ontology scores (Equal Weighted)

Quality	Mean	Std. Deviation	Min	Max
Syntactic	.67	.11	.56	.85
Lawfulness	.97	.04	.91	1
Richness	.36	.19	.15	.69
Semantic	.83	.09	.69	.99
Interpretability	.80	.31	.1	1
Consistency	.73	.25	.37	1
Clarity	1	.01	.98	1
Pragmatic	.14	.26	5.98E-04	.52
Comprehensiveness	.14	.26	5.98E-04	.52
Social	.14	.36	0	.01
History	.14	.36	0	.01
**Overall Score**	**.45**	**.10**	**.31**	**.59**

### Drug Ontologies

We reviewed the list of available biomedical ontologies that were drug-related for selection in our study. The list below are the drug ontologies used:

- RxNORM [14]
- VANDF (Veterans Health Administration National Drug File) [15]
- DRON (Drug Ontology) [16]
- DINTO (Drug-Drug Interaction Ontology) [17]
- DIKB (Drug Interaction Knowledgebase) [18]
- VO (Vaccine Ontology) [19]
- PVOnto (Pharmacovigilance Ontology) [20]

The National Drug Data File, the National Drug File – Reference Terminology, and Master Drug Data Base Clinical Drugs were not included in our experiment due unavailability of a downloadable file for testing.

The study utilized the latest version of OWL-API v4.2.3 [21], MIT JWI v2.4 (for word senses) [22], apache-commons-lang v3.4 [23], and minimal-json v0.9.4 [24] to develop Java software code to calculate the scores. For each of the downloaded ontologies, we collected scores from the software and recorded the values. Scores that relied on total times accessed and the number of classes, instances, and properties were collected from NCBO’s RESTful API.

## Results

The results are detailed in the subsequents sub-sections. Certain scores were neglected due to lack of resources to calculate them (*authority*, *relevancy*, and *accuracy*). Equal weighted (EW) evaluation scoring was used (6). *Pragmatic* score was simply the *comprehensiveness* due to lack of resources to calculate *accuracy* and *relevancy*, and the *social* score was only the *history* score for the same reasons described.

6 $$\begin{array}{@{}rcl@{}} Q_{EW}\! =\! (0.25*S)\,+\,(0.25\! *\! P)\! +\! (0.25\! *\! E)\,+\, (0.25*O) \end{array} $$

### NCBO Bioportal Score (Sample size = 64)

Table 1 depicts the values resulting from the arithmetic mean of the evaluation scores for the top 64 viewed ontologies from September 2015. The mean for the overall quality score for the sample amounted to 0.39 (*μ*=0.05). To calculate the *comprehensiveness* score which required knowing the number of classes, instances, and properties, we tallied a total of 1,277,993, and a total accessed (for the *history* score) at 152,424 based on the entire set, through September 2015.

*Semantic* quality, from the sample set appeared to be strongest with 0.88, and the weakest aspect appeared to be *social* and *pragmatic* quality. At a more granular level, *clarity* which measured ambiguity of terms and labels revealed a score of 0.96. *Lawfulness* which measured adherence to ontology standards was also high at 0.92.

### Drug Ontology Scoring

#### Equal weighted scores

Table 2 provides data from equal weighted evaluation scoring for the set of drug ontologies we assessed. 0.45 (*σ*=0.10) is the average mean for the 7 drug ontologies. The total number of classes, instances, and properties used to derive the *comprehensiveness* score was 169,862, and the total number of times the ontology was accessed was 351,616. This was used to formulate the *history* score (*social*).

From the results and similar to the previous sample set, *semantic* quality was the prominent with 0.83 (0.88 for NCBO). For the sub-scores, *clarity* and *lawfulness* both exhibited high ratings, 1 and 0.97 respectively.

#### Drug ontology-influenced modulated scores

From the scores generated earlier, we devised a method to customize the metrics to accommodate the set of drug ontologies by modifying the weights. The *semantic*, *pragmatic*, *syntactic*, and *social* were 0.83, 0.14, 0.67, and 0.14. The values were converted proportionally to give weights for *semantic*, *pragmatic*, *syntactic*, and *social* (0.46, 0.08, 0.38, and 0.08). With the new values, we replaced the weights to attain (7), and recalculated our data. Table 3 shows the results from the modulated scoring with each drug ontology with the unmodified scores, *Q*_*mod*_ and *Q*_*EW*_ respectively. These values were the overall final scores for *Q*_*mod*_ and *Q*_*EW*_.

7 $$\begin{array}{@{}rcl@{}} Q_{mod}\! =\! (0.38\! *\! S)\! +\! (0.08\! *\! P)\! +\! (0.46\! *\! E)\! +\! (0.08\! *\! O) \end{array} $$

**Table 3:** Examination of the weighted scores

	*Q*_*EW*_	*Q*_*mod*_	Diff	S+E	P+O
RxNORM	0.64	0.69	0.05	0.70	0.11
DIKB	0.44	0.75	0.31	0.88	0.00
DINTO	0.41	0.69	0.28	0.81	0.01
PVOnto	0.38	0.66	0.28	0.76	0.00
VANDF	0.35	0.57	0.22	0.67	0.02
VO	0.37	0.63	0.26	0.74	0.00
DRON	0.53	0.64	0.11	0.70	0.35
***μ*** **(** ***σ*** **)**	**0.45 (0.10)**	**0.66 (0.05)**	**0.21**	**0.75**	**0.07**

From Table 3, RxNORM under the equal weighted evaluation metric amounted to 0.64 (6) and the modulated score of 0.69 (7). Similar increases as a result of the modulated scoring produced the same result for the other drug ontologies. The means of the overall scores were 0.45 and 0.66 (before and after, respectively).

## Discussion

In this section, the paper will discuss how the equal weighted drug ontologies compared to the sample set of NCBO ontologies (also equal weighted). The purpose is to assess how an ontology or a group of specific type of ontologies align with the quality of biomedical ontologies. Also, this section will compare the equal weighted scoring of drug ontologies and the modulated scoring of drug ontologies. This will assess whether the modulated metrics represented the drug ontologies better than the equal weighted version. Lastly, the paper will further examine each individual scores of each drug ontology.

### Comparative results with NCBO sample data

When calculating the *comprehensiveness* and *history* score, we utilized the total number of ontological elements and total times accessed relative to the set they belong to. Therefore, we will neglected comparison between *pragmatic* and *social* and focused on the other scores between the NCBO sample and the drug ontology scores, both of which were equal weighted. Without the aforementioned scores, the overall average mean of the final quality score were both 0.38, keeping the weights at 0.25 for *syntactic* and *semantic*. Closer inspection of the values between the two tables (Tables 1 and 2) reveal some close alignment with the greater body with NCBO ontologies from the sample. *Syntactic* and its related sub-scores resemble the same values, however, the *semantic* quality scores might have some deviation. The consistency sub-score, which scores an ontology’s term uniformity (minimal duplication of terms and labels), appear to be distinguishable with NCBO sample aggregate (0.73 to 0.84). This could possible reveal that some drug ontologies may have some duplicated labels, and may have to resolve those duplication if the ontology is to be deemed consistent in its domain space within the semiotic framework. Since we are utilizing a sample set from NCBO, any conclusion drawn should be cautiously considered. Nonetheless, one way of evaluating on ontology, particularly one that is under-development is to compare the scores with the greater body of biomedical ontologies.

### Comparative results with modulated drug ontology scores

We compared the overall quality scores (6) and the analogous modulated overall quality score (7) for each of the drug ontologies (Table 3). With the equal weighted approach, RxNORM and DRON produced higher quality scores (0.64 and 0.53). Examining their respective scores, specifically looking at *S* (*syntactic*) and *E* (*semantic*) together (*S*+*E*), we noted that both RxNORM and DRON were below average compared to other drug ontologies (Table 3). However, looking at just *P* (*pragmatic*) and *O* (*social*) together (*P*+*O*), RxNORM and DRON score above average, while the rest of the drug ontologies rates below average. So the relatively high overall score of RxNORM and DRON was mainly due to their advantage of being accessed more and being more “comprehensive” than the other drug ontologies, which alluded to some “unfairness” in the equal weighted metrics.

Focusing the attention on the modulated weighted scores for the drug ontologies, DIKB ended being the better quality drug ontology over RxNORM with an overall score of 0.75 than RxNORM’s 0.69. DINTO also yielded a score of 0.69. All of the drug ontologies exhibited an increase (*μ*=0.21,*σ*=0.1), but RxNORM and DRON produced the smallest gains (0.05 and 0.11). Because the modulated scoring increased the weights for *syntactic* and *semantic*, where the quality scores of DIKB, DINTO, and PVOnto exhibited relatively high values, DIKB, DINTO, and PVOnto reported the largest gains. Also with the lessen weights for *pragmatic* and *social*, RxNORM and DRON did not have the high quality score that it had previously.

**Fig. 1:**
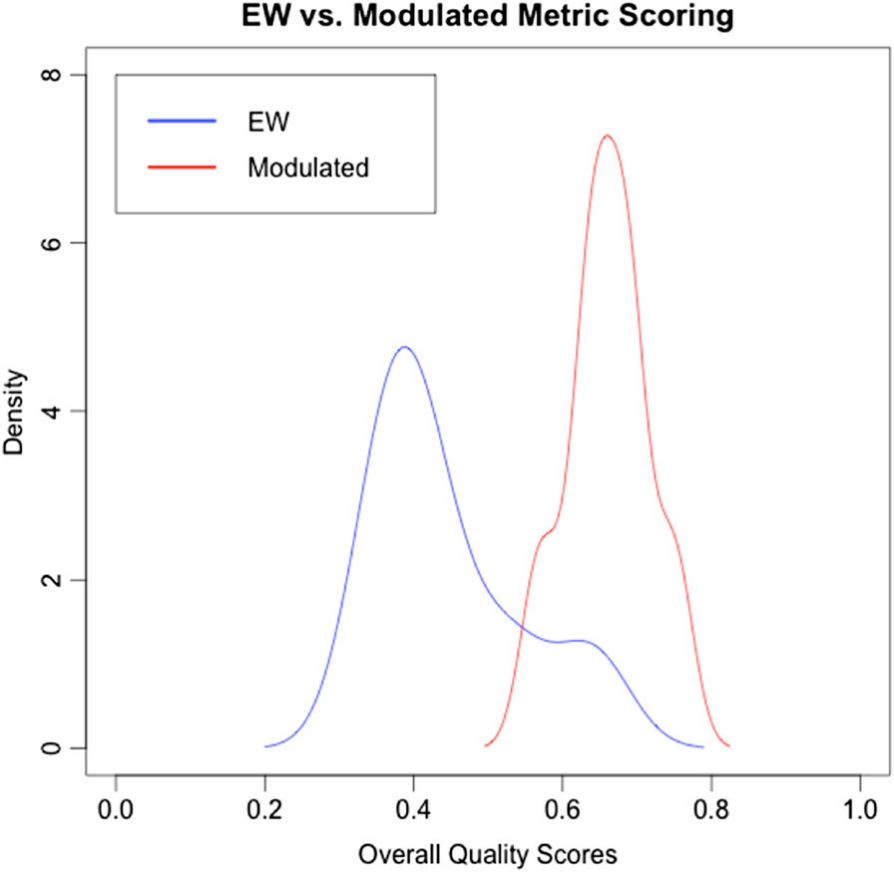
Density plot of overall quality scores

The average for the entire drug ontology for the equal weighted metrics was 0.45 (*σ*=0.10) and for modulated weighted was 0.66 (*σ*=0.05). Figure 1 shows a simple histogram of both the equal weighted and modulated weighted overall score. In general, the modulated metric that we formulated, what could be, a more faithful and authentic scoring for drug ontologies. The impact of this specific effort could provide direction for knowledge engineers to utilize the semiotic framework to tailor it for specific groups of ontologies. Also, it could be a start towards a standard metric for any new drug ontologies under-development or introduced.

### Individual drug ontology scores

For each of the drug ontologies, Table 4 provides an examination of individual scores and sub-scores. The following subsections will discuss some observations of these values.

**Table 4:** Individual drug ontology quality scores

	SL	SR	S	EI	EC	EA	E	PO	P	OH	O	*Q*_*EW*_	*Q*_*mod*_
RxNORM	0.91	0.21	0.56	0.97	0.54	1.00	0.83	0.22	0.22	0.96	0.96	0.64	0.69
DIKB	1.00	0.67	0.84	0.96	0.87	0.98	0.93	0.00	0.00	0.00	0.00	0.44	0.75
DINTO	1.00	0.49	0.75	0.80	0.88	1.00	0.88	0.03	0.03	0.00	0.00	0.41	0.69
PVOnto	1.00	0.15	0.58	0.93	0.96	0.99	0.95	0.00	0.00	0.00	0.00	0.38	0.66
VANDF	0.91	0.21	0.56	0.96	0.37	1.00	0.77	0.05	0.05	0.03	0.03	0.35	0.57
VO	1.00	0.38	0.69	0.89	0.51	1.00	0.79	0.00	0.00	0.00	0.00	0.37	0.63
DRON	0.96	0.44	0.70	0.10	0.98	1.00	0.69	0.71	0.71	0.01	0.01	0.53	0.64
**Mean**	**0.97**	**0.36**	**0.67**	**0.80**	**0.73**	**1.00**	**0.83**	**0.14**	**0.14**	**0.14**	**0.14**	**0.45**	**0.66**
**Median**	**1.00**	**0.38**	**0.69**	**0.93**	**0.87**	**1.00**	**0.83**	**0.03**	**0.03**	**0.00**	**0.00**	**0.41**	**0.66**
**St Dev**	**0.04**	**0.19**	**0.11**	**0.31**	**0.25**	**0.01**	**0.09**	**0.26**	**0.26**	**0.36**	**0.36**	**0.10**	**0.05**
**Min**	**0.91**	**0.15**	**0.56**	**0.10**	**0.37**	**0.98**	**0.69**	**0.00**	**0.00**	**0.00**	**0.00**	**0.35**	**0.57**
**Max**	**1.00**	**0.67**	**0.84**	**0.97**	**0.98**	**1.00**	**0.95**	**0.71**	**0.71**	**0.96**	**0.96**	**0.64**	**0.75**

#### Syntactic level

DIKB, DINTO, and DRON exhibited strong *semantic* quality (*S*) as evident by the high scores. Looking at both DIKB and DINTO’s *richness* (*S* *R*) and *syntactic* (*S* *L*) sub-scores both rated very high, revealing low ontological violations and utilized more ontological features. DRON’s *richness* score was below the average, yet the average was particularly high. The strength of DRON was due to the utilization of many ontological features. Both RxNORM and VANDF rated below average for *syntactic* quality, and both had the lowest *richness* and *syntactic*, indicating relatively lower than average use of ontological features and more standards violations.

Because of the very high *syntactic* (*S* *L*) score, there was a high standard of adherence to syntactical aspect with drug ontologies. *Richness* (*S* *R*) varied among them as the scores were differed greatly where half preformed better than average. Observationally, the drug ontologies that exhibited stronger syntactic richness tend to have higher *semantic* (*S*) score.

#### Semantic level

Examining the *semantic* quality, DIKB, DINTO, and PVOnto displayed the highest scores. All three denote better than average sub-scores for *interpretability* (*E* *I*), *consistency* (*E* *C*), and *clarity* (*E* *A*) – ontological terms’ expressiveness, uniqueness, and ambiguity. DINTO assessed less ambiguity, DIKB’s unique trait appear to be *interpretability*, and PVOnto strong point was the consistent usage of terms and labels. VANDF rated lower than average and lowest of the group for *semantic* quality. This was due to consistency being drastically lower, even though it exhibited expressive terms and less ambiguity of the terms.

Overall, *clarity* is exemplary among the drug ontologies, indicating less ambiguity among the terms, however they vary with *consistency* and *interpretability*. Drug ontologies could benefit from better selection of terms and finding terms with better expressiveness (terms with at least one word sense).

#### Pragmatic level

Noted earlier, *pragmatic* (*P*) score was limited by the use of *comprehensiveness* (*P* *O*) sub-score. To reiterate, *comprehensiveness* was determined by the number classes, instances, and properties over the total of those elements in a set. Both DRON and RxNORM exhibited higher than the median score for (*P*). DRON had substantially prominent *pragmatic* score with 0.71 (*μ*=0.14,*σ*=0.26). Scores that denoted 0.00 had values very low to display to two significant digits. Prolific drug ontologies tended to be large in size and scope.

#### Social level

Similar to *pragmatic* (*P*), the *social* (*O*) score was determined by one sub-score – *history* (*O* *H*). *Social* measures the ranking of the ontology among the community. RxNORM indicated a very prominent score of 0.96 (*μ*=0.14,*σ*=0.36). With a median among them being 0, most of the drug ontologies compared to RxNORM did not have same level access or popularity. It is difficult to determine ways to improve *history* (number of times of accessed) of ontologies that are not as prolific. However, if community ranking of an ontology is important to a researcher or developer, this score would be an interesting factor to consider in any decision making for biomedical ontology selection or usage.

## Limitations and Future Direction

This study utilized the Burton-Jones, et al. semiotic evaluation metric suite to assess NCBO ontologies, and drug-related ontologies. Despite our efforts in revealing new findings about drug ontologies and establishing a method to tailor evaluation for a set of ontologies, some of what was presented had some limitations.

One of them is the sample set of NCBO ontologies. In the future, we would ideally like to have a larger body of ontologies from NCBO to generate a more representative score for comparative purposes with other ontologies or a group of ontologies, as we have shown in this study. With a larger set, it is also possible to look at other factors that can be considered for evaluation, like breadth, number of children nodes, etc. Also, a few of the scores we could not produce values due to lack of time and human resources to preform reviews for scores like *accuracy* or *relevancy*. However, the benefit of the semiotic framework for ontology evaluation is the openness to customize the metric to suit certain situations, like the lack of subject matter experts.

Initially, we investigated the option for an “automated” approach to determine appropriate weights for the ontologies. However, we deduced that tailoring the weights is subjective, and that an automated approach would likely provide weights independently of a priori knowledge. Yet one possibility that was considered, and perhaps a future possibility, was investigating the use of genetic programming algorithms [25] to approximate weights for the drug ontologies, and then apply k-fold validation to establish if the suggested weights are useful. Supervised learning or other related approaches are potential options.

### SEMS (Semiotic Evaluation Metric Suite) aka “Ontokeeper”

**Fig. 2:**
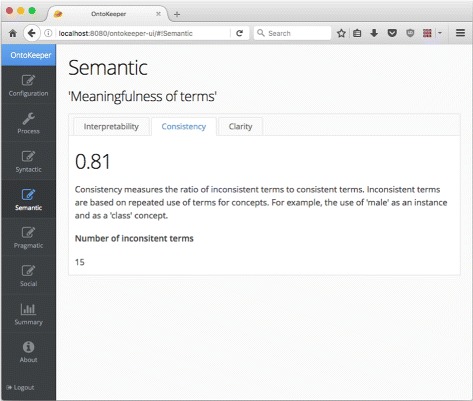
Ontokeeper screenshot

Another direction we are engaged is to develop a front-end tool for users to evaluate ontologies very quickly, and also to have some suggested ideas for users to improve the ontology based on the scores [26]. The prototype web-based tool was called SEMS (Semiotic Evaluation Metric Suite), now called “Ontokeeper”, which supports most of the automated score generation, and will facilitate the collection of feedback from subject matter experts to assist in the calculation of the accuracy score. Figure 2 shows a sample screenshot of the updated version of Ontokeeper.

## Conclusion

Using a semiotic framework for ontology evaluation, this paper demonstrated a tailored metric that closely approximated the quality of a set of NCBO drug ontologies. The scores and sub-scores from examination indicated that NCBO drug ontologies could improve with greater use of syntactic ontological features, better selection of terms and terms with expressive quality, and perhaps improve consistency among the terms and labels. Through the use of a multidimensional metric-based approach, our efforts may be one of several promising directions for biomedical ontology evaluation that needs further investigation.
